# Changing youth behaviour in South Africa

**DOI:** 10.4102/hsag.v25i0.1031

**Published:** 2020-01-29

**Authors:** Nelisiwe Khuzwayo, Myra Taylor, Catherine Connolly

**Affiliations:** 1 Discipline of Rural Health, School of Nursing and Public Health, College of Health Science, University of KwaZulu-Natal, Durban, South Africa

**Keywords:** carried weapons, sexual behaviours, alcohol abuse, students, youth behaviour

## Abstract

**Background:**

Youth behaviour in South Africa continues to be a public health concern. Primary prevention interventions remain a pre-requisite for promotion of improved social and health outcomes.

**Aim:**

The aim of the study was to assess the impact of a behavioural youth risk reduction intervention among grade 10 learners.

**Setting:**

The Study was conducted in KwaZulu-Natal high schools, at UMgungundlovu District Municipality.

**Methods:**

We conducted a cluster randomised controlled trial at 16 KwaZulu-Natal high schools where learners completed a self- administered questionnaire assessing youth risk behaviours. Schools were divided into two arms, intervention and a control arms.

**Results:**

The intervention reduced learners’ reports of carrying of a weapon to school in the past 30 days, but did not significantly reduce other assessed risk behaviours. Although the intervention appeared more likely to reduce learners’ risk behaviours when compared to the control group, such as carrying weapons, risky sexual behaviour and alcohol and drug abuse.

**Conclusion:**

This study was unable to show statistical significance for these outcomes.

## Background

Globally, youth risk behaviour, including risky sexual behaviour, drug abuse and violence, is a public health concern and South Africa is no exception. A range of behaviours place youth – and in the context of this study, South African youth – at risk. Although numerous interventions have been conducted to mitigate risk-taking, young people continue to practise unsafe sex, binge drink and use illicit drugs, and are involved in violence. The latest South African survey on youth risk behaviours reports that adolescents initiate alcohol use prior to the age of 13 years, and that men were more likely than women to use alcohol, engage in binge drinking, to have driven or walked under the influence of liquor and engaged in physical fighting (Burton & Leoschut 2013a; Shisana et al. 2015). Furthermore, in South Africa, although the human immunodeficiency virus (HIV) infection rate is decreasing, youth are severely affected by HIV, violence and unplanned teenage pregnancy (District 2012; National Department Health 2012; Mkhwanazi 2010). It is also widely acknowledged that the high prevalence of HIV and other sexually transmitted infections and rate of teenage pregnancy are fuelled by high-risk behaviours (Harrison et al. 2010a).

Evidence shows that substance abuse has detrimental consequences for youth (Scott-Sheldon et al. 2013; Yach et al. 2015). The results of the South African national violence survey reported that of the 47% learners who smoked marijuana at school, 31% reported witnessing learners who were high and 27% reported knowing learners who were drunk at school (Burton & Leoschut 2013a). This is undesirable in a teaching and learning environment and particularly concerning, as alcohol abuse has been associated with violent behaviour (Peltzer, Davids & Njuho 2011). The prevalence of violence in South African schools has been reported to be high (Schuld 2013). One in four learners reported knowing learners who brought weapons such as firearms, knives and other sharp objects to schools (Burton & Leoschut 2013a; Ward et al. 2012). These learners may carry weapons to initiate or threaten other students, or for self-defence. The prevalence rates of students reported carrying weapons vary across the country (Burton & Leoschut 2013a). Learners from KwaZulu-Natal province were aware of learners who had brought a weapon to school, and 8.2% stated that they were threatened whilst at school (Burton & Leoschut 2013a).

Another concern is the lack of contraceptive use (Bhana et al. 2010; Catalano, Gavin & Markham 2010). In the third South African Youth Risk Behaviour Survey (SAYRB [2011]), learners reported condom use (45.1%) as the main method to prevent pregnancy; this was followed by contraceptive injections (7.0%) and use of pills (4.7%) as other birth control methods. Gender-based violence, especially amongst female students (Harrison et al. 2010a), is a particular concern as some of them are dating older men (Dellar, Dlamini & Karim 2015; Haberland & Rogow 2015). Evidence shows that they are unable to negotiate condom use because their male partners are older and tend to have many casual partners (Onoya et al. 2012; Potgieter et al. 2012).

South Africa has responded through numerous interventions, including condom distribution programmes (Dellar et al. 2015; Prinsloo 2007) and HIV education (Harrison et al. 2010a). There have also been a number of well-publicised HIV awareness campaigns using a variety of media, including Khomanani, Love Life, Soul City and Soul Buddy (Bekker et al. 2015; Chandra-Mouli et al. 2015). The 2012 National Communication Survey on HIV/AIDS evaluated these national campaigns and found that they were having a positive effect, particularly on students aged 15–24 years, with an increase in condom usage, HIV testing, counselling and male circumcision (Peltzer et al. 2012). However, research interventions, which included large trials, have shown mixed results with regard to the reduction of risk behaviour (Harrison et al. 2010a). Students aged 15–24 years in South Africa continue to have poor health outcomes because of their high-risk behaviour (Harrison et al. 2010a; National Department Health 2012). There is an urgent need to find interventions that may prevent and reduce youth risk behaviour.

We thus developed, implemented and evaluated a context-based behavioural risk-reduction intervention in uMgungundlovu District Municipality, KwaZulu-Natal province, aimed at reducing risky sexual behaviour, use of alcohol and other drugs and violence enacted by learners.

## Purpose of the research

The purpose of this study was to assess the effects of a behavioural risk-reduction intervention on sexual risk behaviours, substance abuse and violence-related behaviours of grade 10 learners assigned to receive a behavioural youth risk-reduction intervention, compared to those who did not receive such an intervention.

## Methods

The study was conducted in 16 public co-educational high schools in uMgungundlovu District Municipality, South Africa, from 2014 to 2015. This district comprises seven local municipalities (Impendle, Mkhambathini, Mpofana, Msunduzi, Richmond, uMngeni and uMshwathi), six of which were selected. The Office of the Premier of KwaZulu-Natal requested to exclude Msunduzi local municipality as it felt that the said municipality was already saturated with projects.

We invited high schools to participate in the behavioural risk-reduction trial. Only public high schools were eligible because of the existence of the Operation Sukuma Sakhe facilitators and we randomly selected 16 of the 45 high public schools in the district (Creswell 2013). To ensure comparability of intervention and control groups, schools were stratified and pairs of schools were randomly selected from the same stratum, one randomly allocated to intervention and another to control (Creswell 2013).

## Sample size

Owing to financial constraints, this study focused only on grade 10 learners. The sample size of 2000 grade 10 students from 16 schools was calculated to detect a 19% reduction in the proportion of students engaging in risky behaviours between intervention and control groups. We set a study power of 80% and a significance of 95% probability. A design effect of 7 was included in the calculation to account for the randomised clustered design of the trial.

## Instrumentation

A questionnaire based on the SAYRBS comprising the following variables was used and piloted prior to being completed by the learners: demographic profile, violence-related behaviours, substance abuse, sexual-related behaviours, circumcision, HIV and AIDS. A pilot study was conducted amongst grade 10 learners in schools which did not form part of the main study, and 22 learners completed the questionnaires.

Demographic profile: These questions comprised age, sex, living arrangements and information about the head of the household (see Table 1).

**TABLE 1:** Demographic profile of student participants (*n* = 1558).

Variable	Intervention		Control		*p*
	*n*	%	*n*	%	
**Age group (years)**					
13–15	159	19	232	32	< 0.001
16–17	404	49	316	43	-
18–23	260	32	181	25	-
Total	823	-	729	-	-
**Sex**					
Male	402	49	364	50	0.7
Female	421	51	365	50	-
Total	823	-	729	-	-
**Municipal**					
Richmond	193	23	135	19	< 0.001
Mpofana	132	16	85	12	-
Mshwathi	190	23	94	13	-
Impendle	109	13	174	24	-
Umngeni	65	8	119	16	-
Mkhambathini	134	16	122	17	-
Total	823	-	729	-	-
**Whom do you live with?**					
Both parents	206	25	204	28	0.4
Father	46	6	34	5	-
Mother	298	36	245	34	-
Other	272	33	245	34	-
Total	822	-	728	-	-
**Highest grade of head of household**					
Never/primary	216	26	162	22	< 0.001
High school	505	62	423	58	-
Post-high school	98	12	143	20	-
Total	819	-	728	-	-
**Where do you live?**					
House	724	90	621	89	0.2
Rented room	16	2	19	3	-
Employer	10	1	19	3	-
Informal settlement	23	3	14	2	-
Other	28	3	25	4	-
Total	801	-	698	-	-
**Type of work of head of household**					
Professional	120	15	167	23	< 0.001
Skilled	116	14	119	16	-
Unskilled	222	27	191	26	-
Not working	362	44	249	34	-
Total	820	-	726	-	-

To investigate the risk behaviour of participants, the SAYRBS was used. The SAYRBS is a school-based survey used to monitor priority health risk behaviours that contribute to the leading causes of death, disability and social problems amongst youth.

Violence-related behaviours: These were adapted from the SAYRBS. The measures of whether students carried a weapon and a gun were as follows: (1) ‘During the past 30 days, how many days did you carry a weapon such as a gun, knife or club on school property?’ and (2) ‘During the past 30 days, on how many days did you carry a gun?’ These were scored as 0 = no days, 1 = 1–2 days and 2 = 3–4 days.

Substance abuse: The measures of cigarette, alcohol and marijuana use were limited to the past one month. (Marijuana is very prevalent and easily available in the local communities where the schools are situated.) Time, frequency and quantity were measured. Questions included were as follows: (1) ‘On how many days did you smoke a cigarette?’ (1 = 0, 2 = 1–2 and 3 = 3–30 days), (2) ‘How many cigarettes did you smoke on an average per day?’ (1 = 0 cigarettes, 2 = 1 cigarette and 3 = 2–20+ cigarettes), (3) ‘How often in the past one month did you have at least one drink of alcohol (e.g. beer wine and brandy)?’, (4) ‘How often in the past one month did you have five or more drinks of alcohol?’ (1 = 0 days, 2 = 1–2 days and 3 = 3–30 days), (5) ‘How many number of drinks did you have in a row?’ (1 = 0 drinks, 2 = 1–3 drinks and 3 = 4–30 drinks) and (6) ‘How many times did you smoke marijuana on average per day?’ (1 = 0 times, 2 = 1–9 times and 3 = 10–40+ times). The questionnaire used the local term *dagga* instead of marijuana.

Sexual-related behaviours: Having high-risk sexual intercourse was measured by the following two items: (1) ‘Did you drink alcohol or use drugs before you had sexual intercourse the last time you were having sex?’ (1 = yes and 2 = no) and (2) ‘Did you or your partner use a condom during sexual intercourse the last time?’ (1 = yes and 2 = no).

The following questions were added at the follow-up after 4 months in order to investigate the effectiveness of the then current HIV testing campaign and circumcision for HIV and AIDS. The questions were adapted from the SAYRBS measures of HIV testing and perceptions and comprised the following: (1) ‘Have you ever been tested for HIV?’ (1 = yes or 2 = no). (2) ‘Do you think you might be HIV-positive?’ (1 = yes, 2 = no and 3 = don’t know). (3) ‘When it comes to HIV do you feel you are?’ (1 = at risk of getting HIV, 2 = somewhat at risk, 3 = not at risk and 4 = don’t know).

Circumcision: Having been circumcised was measured by the following item: ‘have you ever been circumcised?’ (1 = yes and 2 = no).

HIV and AIDS: The measures of HIV testing and perceptions comprised the following: (1) ‘Have you ever been tested for HIV?’ (1 = yes or 2 = no), (2) ‘Do you think you might be HIV-positive?’ (1 = yes, 2 = no and 3 = don’t know) and (3) ‘When it comes to HIV, do you feel you are? (1 = at risk of getting HIV, 2 = somewhat at risk, 3 = not at risk and 4 = don’t know).

## Procedures

All 16 participating schools were allocated to the intervention and control groups using stratified random sampling. General information sessions about the study were facilitated for the principals, school governing bodies and all grade 10 students in all 16 high schools. This process provided an opportunity for the recruitment of potential participants. Information sheets and informed consent forms were sent to parents or guardians of all grade 10 students and were collected before the actual enrolment process. During enrolment, all the learners who were willing to participate signed informed consent and assent forms. This study was approved by the University of KwaZulu-Natal ethics committee (BREC REF: BE342/14), and the Department of Education gave permission to conduct the study in the school. Ethical issues such as confidentiality, anonymity and voluntary participation were discussed with parents or guardians and students during the enrolment process. All students in both arms of the intervention completed a baseline and follow-up survey 4 months after the intervention using a questionnaire that was completed in the classroom. About five students did not participate because of the non-availability of parental consents, and there were no other refusals in this study.

## Intervention arm

After each school completed enrolment and the baseline survey, a behavioural risk-reduction intervention was implemented if a school was in the intervention group. Two facilitators per school delivered sessions, which took place during the life orientation (LO) periods. The duration of the sessions lasted from 45 min to 1 h and comprised the following topics: ‘knowing yourself’, ‘peer pressure’, ‘decision-making’, ‘healthy and unhealthy relationships’, ‘contraceptives’, ‘teenage pregnancy’, ‘condom use’, ‘HIV/AIDS and STI prevention’, ‘alcohol and drug abuse’, ‘violence and gender-based violence’, ‘child support grant’, ‘human rights’ and ‘responsibilities in sexual health’. The sessions were designed to include brainstorming group discussions, role-plays, reflections and demonstrations. All the eight schools of intervention group agreed for two sessions per week and the intervention was implemented in all these schools within 2 months. We assessed the process of implementation of the intervention by requesting that the facilitators record each session by giving students an attendance register to sign for each session.

## Control arm

There was no intervention in the control schools. They continued with their LO Learning Area, which is a compulsory learning area for all learners attending public schools.

## Statistical analysis and evaluation

We matched pre- and post-questionnaires by student’s Identification (ID). The questionnaires were coded to ensure confidentiality. We recorded question responses using the Likert scale, categorised into three groups: no risk, moderate risk and high risk at baseline. The intervention effect was measured by the difference between learners’ responses on the pre-test and post-test questions. This difference was dichotomised into positive and negative behaviour (showing no change or an increase in risk behaviour). A generalised estimating equation (GEE) model was used to compare the groups (Creswell 2013). This model adjusts for the possible correlation of students within schools and baseline characteristics, such as age. Significance was set at *p* < 0.05 for all the analyses. We used STATA 13 statistical software to analyse the data.

### Ethical consideration

Ethical approval to conduct the study was obtained from the University of KwaZulu-Natal’s Biomedical Research Ethics Committee in South Africa (BREC REF: BE 342/14).

## Results and findings

**FIGURE 1:**
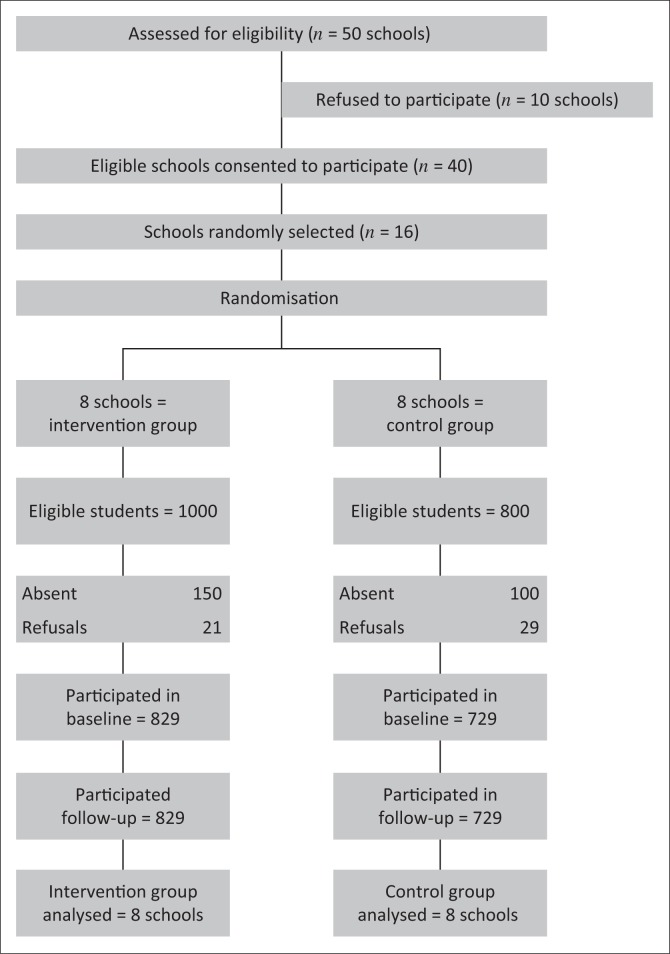
Randomised controlled trial study flow (*n* = 1558).

A total of 16 high schools and 1558 grade 10 learners participated in the trial, with eight schools per group (see Figure 1). All were analysed at follow-up.

Table 1 shows the characteristics of participants. The ages of the learners ranged from 13 to 23 years. The old age of some learners was a result of KwaZulu-Natal’s policy not to exclude older learners from rural schools. Students from the intervention group were older than the control group (*p* < 0.001). Male and female learners were equally represented. More than one-third of learners (35%) were residing with their mothers and extended families, whilst only 28% were living with both parents (*p* = 0.40). Over half (928) of the heads of the households had been to high school, but surprisingly household heads in the control group were more likely to have post-high school qualifications and be professionals (*p* < 0.001).

Table 2 shows the baseline prevalence of risk behaviour in intervention and control groups. Measures were confined to 30 days. Of the learners in the intervention group, 115 (14%) carried a weapon and 61 (8%) carried a gun, and in the control group 73 (10%) and 36 (5%) had carried a weapon and a gun, respectively. One third of the learners reported alcohol consumption, with 13% in the intervention and 12% in the control groups reported having binge drinking (five or more glasses of alcohol at one time). Of the learners in both groups (intervention and control), 268 (33%) versus 220 (31%) reported smoking cigarettes and 126 (16%) versus 107 (15%) had smoked marijuana at least once in the past 1 month, respectively.

**TABLE 2:** Prevalence of students’ risk behaviours for previous 30 days reported at baseline between intervention and control groups.

Variable	Intervention		control	
	*n*	%	*n*	%
**Carry a weapon**				
0 days	694	86	648	90
1–4 days	115	14	73	10
**Carry a gun**				
0 days	748	92	685	95
1–4 days	61	8	36	5
**Smoked cigarettes**				
0 days	572	71	526	73
1–30 days	237	29	195	27
**Cigarettes smoked/day**				
0	537	67	500	69
1–20 cigarettes	268	33	220	31
**Drank alcohol**				
0 days	459	57	436	60
1–30 days	351	43	286	40
**Drank 5+ alcohol**				
0 days	555	68	516	72
1–30 days	258	32	205	28
**Drinks in a row**				
0 drinks	467	58	444	61
1 to 10+ drinks	345	42	279	39
**Marijuana times**				
0 times	683	84	618	85
1–40+ times	126	16	107	15
**Alcohol before sex**				
Yes	42	14	34	14
No	263	86	203	86
**Condom before sex**				
Yes	193	64	123	53
No	107	36	109	47
**HIV test**				
Tested	481	60	368	51
Not tested/unsure	317	40	348	49

A quarter of the sexually active learners, 42 (14%) versus 34 (14%) reported drinking alcohol before having sex and 40% of the learners had not used a condom at their last sexual intercourse. Of the learners, 60% in the intervention group and 51% in the control group reported testing for HIV in the past 1 month.

### Behavioural change

**FIGURE 2:**
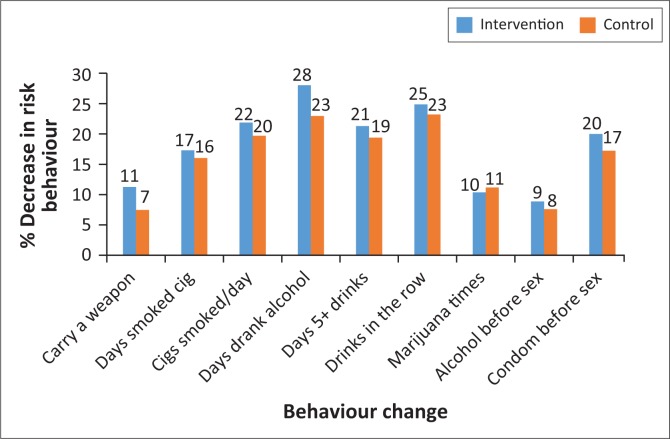
Decrease in high school students’ risk behaviours between intervention and control groups.

There was a decrease of 11% between baseline and follow-up amongst the learners in the intervention group reporting carrying of a weapon during the previous 30 days compared to the 7% decrease in the control group (see Figure 2). Although there was a decrease in the use of tobacco and alcohol in the intervention group, this was not statistically significant. The 10% decrease in the use of marijuana in the intervention group was similar to that in the control group. Similarly, decrease in the use of alcohol before sex was similar to that of the control group (9% vs. 8%). Condom use before sex increased substantially by 20% in the intervention compared to 17% in the control group, but this was not statistically significant (see Table 3).

**TABLE 3:** Comparison of changes in the odds of students’ risk behaviours in the intervention and control groups 4 months later.

Variable	Intervention decrease		Control decease		Unadjusted			Adjusted		
	*n*	Total	*n*	Total	OR	95% CI	*p*	OR	95% CI	*p*
Carry a weapon	91	809	54	721	1.6	1.1–2.5	0.02	1.8	1.2–2.6	0.002
Carry a gun	49	809	32	721	1.4	0.9–2.2	0.2	1.5	0.9–2.5	0.09
Days smoked cigarette	140	809	116	723	1.1	0.7–1.5	0.7	1.1	0.8–1.4	0.6
Cigarettes smoked/day	176	805	142	720	1.1	0.8–1.6	0.6	1.1	0.9–1.4	0.4
Day drank alcohol	227	810	165	722	1.2	0.8–1.8	0.3	1.2	0.9–1.6	0.3
Days 5+ drinks	173	813	139	721	1.1	0.8–1.5	0.5	1.0	0.8–1.3	0.9
Drinks in a row	202	812	167	723	1.1	0.8–1.6	0.6	1.0	0.8–1.3	0.9
Marijuana times	84	809	81	725	0.9	0.5–1.7	0.8	0.9	0.6–1.3	0.5
Alcohol before sex	27	305	18	237	1.2	0.6–2.2	0.6	1.3	0.6–2.6	0.5
Condom before sex	60	300	40	232	1.2	0.7–1.9	0.5	1.1	0.6–1.8	0.8

OR, odds ratio; CI, confidence interval.

The odds of learners in the intervention group not carrying a weapon in the previous 30 days were one and a half times greater than that of those in the control group (adjusted odds ratio [OR] 1.8, 95% confidence interval [CI] 1.2–2.6). There was also a trend towards a reduction in carrying a gun (OR 1.5, 95% CI 0.9–2.5).

There were no statistically significant differences between the groups for smoking (OR 1.1, 95% CI 0.7–1.4), alcohol consumption (OR 0.2, 95% CI 0.9–1.6) and binge drinking (OR 1.0, 95% CI 0.8–1.3). Use of alcohol before sex (OR 1.3, 95% CI 0.6–2.6) decreased and use of condom at last sexual intercourse increased (OR 1.1, 95% CI: 0.6–1.8) in both groups.

### HIV and/or AIDS and circumcision

The prevalence of HIV testing by learners in the post-survey was 530 (66%) for the intervention group and 433 (59%) for the control group. Although the results are not statistically significant, we found that more learners in the intervention group (152; 48%) compared to the control group (130; 37%) reported having been newly tested for HIV after the intervention had been completed (adjusted OR 1.4, 95% CI 0.9–2.1; *p* = 0.1). A similar finding was for the sexually active learners, where 94 (54%) learners in the intervention group compared to 66 (46%) learners in the control group had tested for HIV (adjusted OR 1.4, 95% CI 0.8–2.1; *p* = 0.1), but this was not statistically significant.

Many of those learners who did not test for HIV did not perceive themselves to be at risk of HIV. Only 16 (6%) versus 19 (7%) of these learners perceived themselves to be at risk or somewhat at risk, namely 21 (8%) in the intervention group versus 30 (10%) in the control group. A number of learners perceived themselves not to be at risk (namely 47 [18%] vs. 68 [24%]), but most learners of the intervention group (174; 67%) and the control group (171; 59%) were unsure whether they were at risk of contracting HIV.

### Circumcision to reduce HIV transmission

The overall prevalence rate of medical male circumcision (MMC) in the post-survey in both groups was 65%. Amongst circumcised learners, there was no significant difference between intervention and control groups’ perceptions as to their risk of being infected with HIV.

### Limitations

The intervention programme did not have the desired effect on learners’ risk behaviours, with the decrease in substance use, and sexual risk behaviours occurring in both intervention and control groups. Although the intervention was intensive (twice weekly), the 2-month period may have been insufficient for the wide and complex range of topics covered. A further limitation was that the learners’ questionnaire responses were self-reports of their behaviours. The process evaluation indicated that no other interventions had taken place in the schools but media access by students was widespread, and this was not monitored and may have influenced the study results. Although all grade 10 students were invited, the proposed sample size of 2000 was not attained.

## Discussion

Since behavioural risk and school violence surveys were conducted in South African schools, violence has been reported to be high (Burton & Leoschut 2013b; Reddy et al. 2013). Our intervention appears to have reduced the number of days students carried weapons to their schools, with the intervention group indicating a significant reduction in the number of learners carrying weapons to school compared to the control group. There have been numerous interventions aiming at reducing the risk behaviours of young people, but most of the studies on the existing interventions, which focus on sexual risk behaviours such as sexuality education, alcohol and drug abuse and gender-based violence, showed mixed results (Harrison et al. 2010b). In South Africa, interventions targeting violent behaviours, such as physical fighting and carrying weapons amongst learners, are limited. In South Africa, the Department of Basic Education has developed an anti-bullying policy to be implemented across public schools, but it is not clear whether it is being implemented as bullying continues to be high amongst high school learners.

The other risk behaviours that were targeted by the study are similar to youth risk behaviours targeted by South African researchers and other countries. However, the context in South Africa differs in that youth seeking their independence and wishing to enjoy themselves are often limited by their social circumstances. South Africa has one of the highest Gini coefficients (Bosch et al. 2010), indicating societal inequality. Worth mentioning is the increase in HIV testing that was found in the present study.

Overall, a general decrease in risky behaviours in both intervention and control groups was observed. The intervention was developed to take into account the context in which youth facilitators from the area were trained to implement the programme in the schools. The envisaged strategy was that their being employed by the KwaZulu-Natal Premier’s Office would enable them to continue to work in the schools to reinforce the programme efforts to reduce students’ risk behaviours. However, in the year that this school programme was implemented, the youth facilitator programme was discontinued for internal budgetary reasons. The intervention programme, although intensive (twice a week), was only for 8 weeks and lacked a follow-up component to reinforce the intervention messages.

We observed that self-regulation through a motivational continuum from extrinsic and intrinsic motivation in students is necessary (Wood, Rowell & Hong 2013). The study afforded students an opportunity to reflect on and internalise their perceptions of their risk and the consequences of anti-social behaviours, such as violence, including their taking necessary measures towards prevention of HIV and STI infections, teenage pregnancy and prevention of alcohol and drug abuse (Bekker et al. 2015).

Changing behaviour is complex and the health promotion messages need to be reinforced so that students are able to personalise these messages and reduce their risk behaviours. Amongst students, condom use has increased from 14% to 60% over the past decades, which, although insufficient, demonstrates that consistent messages could make inroads towards more health-promoting behaviours (Michielsen et al. 2010). Education is compulsory in South Africa and the life skills component of LO curriculum presented in all grades in public schools offers an opportunity for reinforcing key messages as students move up the grades.

## References

[CIT0001] Bekker L.-G., Johnson L., Wallace M. & Hosek S., 2015, ‘Building our youth for the future’, *Journal of the International AIDS Society* 18(251), 1–7. 10.7448/IAS.18.2.20027 PMC4344540 25724512

[CIT0002] Bhana D., Morrell R., Shefer T. & Ngabaza S., 2010, ‘South African teachers’ responses to teenage pregnancy and teenage mothers in schools’, *Culture, Health & Sexuality* 12(8), 871–883. 10.1080/13691058.2010.500398 20665296

[CIT0003] Bosch A., Rossouw J., Claassens T. & Du Plessis B., 2010, ‘A second look at measuring inequality in South Africa: A modified Gini coefficient’, *School of Development Studies Working Paper* 58, University of KwaZulu-Natal, Durban.

[CIT0004] Burton P. & Leoschut L., 2013a, ‘School violence in South Africa: Results of the 2012 national school violence study’, *Centre for Justice and Crime Prevention Monograph Series* 12, Centre for Justice and Crime Prevention, Mombray, Cape Town.

[CIT0005] Burton P. & Leoschut L., 2013b, *School violence in South Africa: Results of the 2012 national school violence study*, Centre for Justice and Crime Prevention, Mowbray, Cape Town.

[CIT0006] Catalano R.F., Gavin L.E. & Markham C.M., 2010, ‘Future directions for positive youth development as a strategy to promote adolescent sexual and reproductive health’, *Journal of Adolescent Health* 46(3), S92–S96. 10.1016/j.jadohealth.2009.12.026 20172463

[CIT0007] Chandra-Mouli V., Svanemyr J., Amin A., Fogstad H., Say L., Girard F. et al., 2015, ‘Twenty years after International Conference on Population and Development: Where are we with adolescent sexual and reproductive health and rights?’ *Journal of Adolescent Health* 56(1), S1–S6. 10.1016/j.jadohealth.2014.09.015 25528975

[CIT0008] Creswell J.W., 2013, *Research design: Qualitative, quantitative, and mixed methods approaches*, Sage, Thousand Oaks, CA.

[CIT0009] Dellar R.C., Dlamini S. & Karim Q.A., 2015, ‘*Adolescent* girls and young women: Key populations for HIV epidemic control’, *HIV and Adolescents: Focus on Young Key Populations* 64. 10.7448/IAS.18.2.19408 PMC4344544 25724504

[CIT0010] District Health Plan, 2012, *District Health Plan, UMgungundlovu Health Ditrict*, Pietermaritzburg, KwaZulu-Natal.

[CIT0011] Haberland N. & Rogow D., 2015, ‘Sexuality education: Emerging trends in evidence and practice’, *Journal of Adolescent Health* 56(1), S15–S21. 10.1016/j.jadohealth.2014.08.013 25528976

[CIT0012] Harrison A., Newell M.-L., Imrie J. & Hoddinott G., 2010a, ‘HIV prevention for South African youth: Which interventions work? A systematic review of current evidence’, *BMC Public Health* 10(1), 1. 20187957 10.1186/1471-2458-10-102 PMC2843660

[CIT0013] Harrison A., Newell M.-L., Imrie J. & Hoddinott G., 2010b, ‘HIV prevention for South African youth: Which interventions work? A systematic review of current evidence’, *BMC Public Health* 10(1), 102. 20187957 10.1186/1471-2458-10-102 PMC2843660

[CIT0014] Michielsen K., Chersich M.F., Luchters S., De Koker P., Van Rossem R. & Temmerman M., 2010, ‘Effectiveness of HIV prevention for youth in sub-Saharan Africa: Systematic review and meta-analysis of randomized and nonrandomized trials’, *AIDS* 24(8), 1193–1202. 10.1097/QAD.0b013e3283384791 20375876

[CIT0015] Mkhwanazi N., 2010, ‘Understanding teenage pregnancy in a post-apartheid South African township’, *Culture, Health & Sexuality* 12(4), 347–358. 10.1080/13691050903491779 20162476

[CIT0016] National Department Health, 2012, *National Strategic Plan on HIV, STIs and TB 2012–2016*, Pretoria.

[CIT0017] Onoya D., Reddy P., Sifunda S., Lang D., Wingood G.M., Van Den Borne B. et al., 2012, ‘Transactional sexual relationships, sexually transmitted infection risk, and condom use among young black women in peri-urban areas of the Western Cape province of South Africa’, *Women’s Health Issues* 22(3), e277–e282. 10.1016/j.whi.2011.11.006 22265179

[CIT0018] Peltzer K., Davids A. & Njuho P., 2011, ‘Alcohol use and problem drinking in South Africa: Findings from a national population-based survey’, *African Journal of Psychiatry* 14(1), 30–37. 10.4314/ajpsy.v14i1.65466 21509408

[CIT0019] Peltzer K., Parker W., Mabaso M., Makonko E., Zuma K. & Ramlagan S., 2012, ‘Impact of national HIV and AIDS communication campaigns in South Africa to reduce HIV risk behaviour’, *The Scientific World Journal* 2012, 384608 10.1100/2012/384608 23213285 PMC3504395

[CIT0020] Potgieter C., Strebel A., Shefer T. & Wagner C., 2012, ‘Taxi “sugar daddies” and taxi queens: Male taxi driver attitudes regarding transactional relationships in the Western Cape, South Africa’, *SAHARA-J: Journal of Social Aspects of HIV/AIDS* 9(4), 192–199. 10.1080/17290376.2012.745286 23234347

[CIT0021] Prinsloo E., 2007, ‘Implementation of life orientation programmes in the new curriculum in South African schools: Perceptions of principals and life orientation teachers’, *South African Journal of Education* 27(1), 155–170.

[CIT0022] Reddy S.P., James S., Sewpaul R., Sifunda S., Ellahebokus A., Kambaran N.S., et al., 2013, ‘Umthente uhlaba usamila: The 3rd South African national youth risk behaviour survey 2011’, South African Research Council, HSRC Press, Cape Town.

[CIT0023] Schuld M., 2013, ‘The prevalence of violence in post-conflict societies: A case study of KwaZulu-Natal, South Africa’, *Journal of Peacebuilding & Development* 8(1), 60–73. 10.1080/15423166.2013.791521

[CIT0024] Scott-Sheldon L.A., Walstrom P., Carey K.B., Johnson B.T., Carey M.P. & Team M.R., 2013, ‘Alcohol use and sexual risk behaviors among individuals infected with HIV: A systematic review and meta-analysis 2012 to early 2013’, *Current HIV/AIDS Reports* 10(4), 314–323. 10.1007/s11904-013-0177-5 24078370 PMC3946916

[CIT0025] Shisana O., Labadarios D., Simbayi L., Onoya D., Zuma K., Jooste S. et al., 2015, ‘South African national HIV prevalence, incidence and behaviour survey’, 2012, HSRC Press, Cape Town 10.2989/16085906.2016.1153491 27002359

[CIT0026] Ward C.L., Artz L., Berg J., Boonzaier F., Crawford-Browne S., Dawes A. et al., 2012, ‘Violence, violence prevention, and safety: A research agenda for South Africa’, *SAMJ: South African Medical Journal* 102(4), 215–218. 10.5330/PSC.n.2013-16.158 22464496

[CIT0027] Wood C., Rowell L. & Hong E., 2013, ‘Academic motivation: Concepts, strategies, and counseling approaches’, *Professional School Counseling* 16(3), 158–171. 10.5330/PSC.n.2013-16.158

[CIT0028] Yach D., Mthembu Z., Sifunda S., Resnicow K., Mbewu A., Sewpaul R. et al., 2015, ‘A decade of tobacco control: The South African case of politics, health policy, health promotion and behaviour change’, *South African Medical Journal* 103(11), 835–840. 10.7196/samj.6910 24148167

